# Outcomes and survival of infants with congenital duodenal obstruction following Kimura procedure with post-anastomosis jejunostomy feeding tube

**DOI:** 10.1186/s12876-021-01679-8

**Published:** 2021-03-04

**Authors:** Munawir Makkadafi, Aditya Rifqi Fauzi, Setya Wandita, Akhmad Makhmudi, Gunadi

**Affiliations:** 1 Pediatric Surgery Division, Department of Surgery, Faculty of Medicine, Public Health and Nursing, Universitas Gadjah Mada/Dr. Sardjito Hospital, Jl. Kesehatan No. 1, Yogyakarta, 55281 Indonesia; 2 Department of Surgery, Faculty of Medicine, Hasanuddin University/RSUP Dr. Tadjuddin Chalid, Makassar, South Sulawesi 90245 Indonesia; 3 Neonatology Division, Department of Child Health, Faculty of Medicine, Public Health and Nursing, Universitas Gadjah Mada/Dr. Sardjito Hospital, Yogyakarta, 55281 Indonesia

**Keywords:** Congenital duodenal obstruction, Enteral feeding, Full oral feeding, Jejunostomy feeding tube, Length of stay, Overall survival

## Abstract

**Background:**

Several modifications of the Kimura procedure for congenital duodenal obstruction (CDO) have been reported, however, their effects on the outcomes show conflicting results.

**Methods:**

We compared the CDO outcomes following the Kimura procedure with and without post-anastomosis jejunostomy feeding tube (JFT).

**Results:**

A total of 52 CDO neonates were involved (JFT: 13 males and 2 females vs. non-JFT: 14 males and 23 females, *p* = 0.0019). Time to full oral feeding was significantly earlier in the JFT than non-JFT group (14 [interquartile range (IQR), 12–15] vs. 17 [IQR, 14–22.5] days; *p* = 0.04). Duration of parenteral nutrition given to infants with CDO after surgery was significantly shorter in the JFT than non-JFT group (12 [IQR, 10–15] vs. 17 [IQR, 13–23] days; *p* = 0.031). Moreover, enteral feeding was significantly earlier in the JFT than non-JFT group (2 [IQR, 1–3.5] vs. 5 [IQR, 4–6] days; *p* = < 0.0001). However, the length of stay following surgery was not significantly different between groups (16 [IQR, 14–22] vs. 20 [IQR, 17–28] days; *p* = 0.22). Also, overall patient survival did not significantly differ between JFT (66.7%) and non-JFT patients (59.5%) (*p* = 0.61).

**Conclusion:**

Jejunostomy feeding tube shows a beneficial effect on the time to full oral feeding, duration of parenteral nutrition and early enteral feeding in neonates with congenital duodenal obstruction after Kimura procedure.

## Background

Congenital duodenal obstruction (CDO) is the most common cause of intestinal obstruction in neonates [1]. Its incidence varies among studies, ranging from 1 in 5000 to 10,000 live births, and it is more common in male infants [2, 3]. More than 50% of patients with CDO are associated with other congenital abnormalities, including Down syndrome, congenital heart disease (CHD), and VACTERL syndrome [2, 4, 5].

Previously, there are several methods for treatment of CDO, such as transmesolic side-to-side duodenojejunostomy and direct duodenoduodenostomy [6, 7]. However, some complications related to anastomosis are noted for those methods [6]. In 1977, Kimura introduced the diamond-shaped side-to-side duodenoduodenostomy with better outcomes for neonates with CDO. Subsequently, these good outcomes were followed by similar findings of other groups [6]. Since then, the Kimura procedure is considered the most preferred surgery for CDO treatment [7].

Several modifications of the Kimura procedure, including use of a post-anastomosis jejunostomy feeding tube (JFT), have been reported, however, their effects on the outcomes show conflicting results [8–11]. This study investigated the comparison of CDO outcomes following Kimura procedure with (JFT group) and without JFT (non-JFT group).

## Methods

### Patient samples

We conducted a retrospective study of infants with CDO at Dr. Sardjito Hospital and its affiliated hospital, in Indonesia, who underwent the Kimura procedure from January 2015–January 2019. The diagnosis of CDO was established according to clinical manifestations, abdominal radiograph, or upper GI series and surgical findings. The Institutional Review Board of the Faculty of Medicine, Public Health and Nursing, Universitas Gadjah Mada/Dr. Sardjito Hospital, Indonesia approved the study beforehand (KE/FK/0811/EC/2018). Informed consent for study participation was obtained from the parents and/or legal guardians of the infants.

### JFT technique, enteral feeding, and discharge criteria

A 3.5Fr soft feeding tube (Terumo, Japan) was applied as a JFT and introduced during the Kimura procedure with a new small stab incision. The location of JFT was approximately 20 cm distal to Treitz's ligament. The JFT remained in situ and simply removed by pulling it out from the jejunum without any second operation after the full oral feeding was achieved. Infants without JFT only had an orogastric tube. Moreover, the JFT or non-JFT procedures were chosen according to the attending pediatric surgeon’ discretion.

The decision to start enteral feeding was made by a neonatologist. It was based on the presence of bowel sounds, no greenish or reddish gastric residual, and the volume of gastric residual was less than 1 mL/kg/day. The feeding protocol after surgery was standardized between groups and applied equally to both groups. The infants were discharged from the hospital if they showed a good general condition, had no complications of the surgery procedure, were capable of oral feeding, and had achieved full oral feeding. All methods were performed in accordance with the relevant guidelines and regulations.

### Statistical analysis

Data were presented as number/percentages and median/mean with interquartile range (IQR). Mann–Whitney U tests were used to evaluate the differences between non-normal distribution variables and Chi-square or Fisher Exact tests were used for analyzing the differences between nominal variables. Log-rank test was applied to compare the infants’ mortality, while Kaplan–Meier curve was utilized to plot the probabilities of infant survival. IBM SPSS Statistics version 16 (SPSS Chicago, USA) was used for statistical analysis.

## Results

### Baseline characteristics

We used ICD-10 Q41.0 code to identify infants with CDO and collected 70 medical records. We excluded 18 subjects due to incomplete medical records. Thus, we further analyzed 52 infants with CDO (Table 1). The analyses involved 52 neonates with CDO after the Kimura procedure (JFT group: 13 males and 2 females vs. non-JFT group: 14 males and 23 females, *p* = 0.0019) (Table 1).

**Table 1:** Baseline characteristics of neonates with CDO after Kimura surgery at Dr. Sardjito Hospital, Indonesia

Characteristics	Total (N = 52)	JFT (N = 15)	Non-JFT (N = 37)	*p* value
	N (%); median (IQR)	N (%); median (IQR)	N (%); median (IQR)	
*Sex*				
Male	27 (51.9%)	13 (86.7%)	14 (37.8%)	0.0019*
Female	25 (48.1%)	2 (13.3%)	23 (62.2%)	
*Antenatal sonography*				
Normal	6 (35.3%)	3 (33.4%)	3 (37.5%)	0.081
Polyhydramnios/ double bubble	11 (64.7%)	6 (66.6%)	5 (62.5%)	
Gestational age (weeks)	37 (36–38)	37 (34–39)	37 (36–38)	0.58
Maternal age (years)	31 (25.25–35)	28 (24.5–31)	31 (27–35)	0.068
Age of neonates at Kimura procedure (days)	8.5 (4–16.75)	6 (1–12)	9 (5–17)	0.103
Birth weight (gram)	2575 (2070–2975)	2600 (2164–2900)	2500 (2148–2900)	0.864
Body weight at Kimura surgery (gram)	2230 (1959–2652)	2320 (2069–2635)	2180 (1956–2590)	0.864

*CDO* congenital duodenal obstruction, *JFT* jejunostomy feeding tube, *IQR* interquartile range

*Significant (*p* < 0.05)

Seventeen neonates underwent an antenatal sonography which showed polyhydramnios or double bubble in eleven infants (Table 1). There were no significant differences of baseline characteristics between the JFT and non-JFT groups, except gender (Table 1).

### Clinical, associated anomalies and surgical findings

There was a significant difference of clinical findings, including epigastric distention and meconium passage, between the JFT and non-JFT groups with *p*-value of 0.024 and 0.03, respectively, while the associated anomalies and type of duodenal obstruction were not significantly different between groups (Table 2). Many of the infants with CDO had Down syndrome (40.4%), followed by CHD (26.9%). Annular pancreas was the most common cause of CDO in our cohort infants (63.4%). Furthermore, duration of surgery in the JFT group was significantly longer than those of the non-JFT group (135 [IQR, 120–165] vs. 120 [105–120] min, *p* = 0.002) (Table 2).

**Table 2:** Clinical, associated anomalies and surgical findings of neonates with CDO following Kimura surgery at Dr. Sardjito Hospital, Indonesia

Characteristics	Total (N = 52)	JFT (N = 15)	Non-JFT (N = 37)	*p* value
	N (%); median (IQR)	N (%); median (IQR)	N (%); median (IQR)	
*Clinical presentation*				
Bilious vomiting	43 (82.7)	12 (80)	31 (83.8)	0.059
Epigastric distention	50 (96.2)	13 (88.2)	37 (100)	0.024*
Meconium passage	46 (88.5)	11 (73.3)	35 (94.5)	0.03*
*Associated anomaly*				
None	23 (44.2)	9 (60)	14 (37.8)	0.11
Down syndrome	21 (40.4)	4 (26.6)	17 (45.9)	0.38
Congenital heart disease	14 (26.9)	3 (20)	11 (29.7)	0.74
Other congenital anomalies (imperforate anus, Hirschsprung disease, Prune-Belly syndrome)	8 (15.4)	2 (13.3)	6 (13.5)	1.0
*Duodenal obstruction type*				
Type 1	12 (23.1)	3 (20)	9 (24.3)	1.0
Stenosis	7 (13.5)	0	7 (18.9)	0.09
Annular pancreas	33 (63.4)	12 (80)	21 (56.8)	0.20
Duration of operation (min)	120 (108.75–135)	135(120–165)	120 (105–120)	0.002*

*CDO* congenial duodenal obstruction, *IQR* interquartile range, *JFT* jejunostomy feeding tube, *min* minutes

*Significant (*p* < 0.05)

### Outcomes of neonates with CDO after Kimura procedure

Time to full oral feeding was significantly earlier in the JFT than non-JFT group (14 [IQR, 12–15] vs. 17 [IQR, 14–22.5] days; *p* = 0.04) (Table 3). Duration of parenteral nutrition given to CDO infants after surgery was significantly shorter in JFT than non-JFT group (12 [IQR, 10–15] vs. 17 [IQR, 13–23] days; *p* = 0.031). Moreover, enteral feeding was significantly earlier in the JFT than non-JFT group (2 [IQR, 1–3.5] vs. 5 [IQR, 4–6] days; *p* = < 0.0001) (Table 3).

**Table 3:** Outcomes of neonates with CDO after Kimura procedure in Dr. Sardjito Hospital, Indonesia

Outcomes	Total (N = 52)	JFT (N = 15)	Non-JFT (N = 37)	*p*-value
	N (%); median (IQR)	N (%); median (IQR)	N (%); median (IQR)	
Full oral feeding (days)	15 (12–20)	14 (12–15)	17 (14–22.5)	0.04*
Duration of nasogastric output (days)	5 (4–7)	5 (3–6)	5 (4–7)	0.59
Length of stay (days)	20 (15.25–25)	16 (14–22)	20 (17–28)	0.22
Duration of parenteral nutrition (days)	15.5 (11–20)	12 (10–15)	17 (13–23)	0.031*
Initial enteral feeding (days)	4.5 (3–6)	2 (1–3.5)	5 (4–6)	0.001*
Weight on discharge (gram)	2460 (2242–2820)	2633 (2560–2820)	2475 (2112–2820)	0.37
*Survival*				
Survived	32 (61.5)	10 (66.7)	22 (59.5)	0.63
Died	20 (38.5)	5 (33.3)	15 (40.5)	0.204
Age at death (days)	11 (5–20.25)	14 (12–23)	10 (5–17)	

*CDO* congenital duodenal obstruction, *JFT* jejunostomy feeding tube, *IQR* interquartile range

*Significant (*p* < 0.05)

**Fig. 1:**
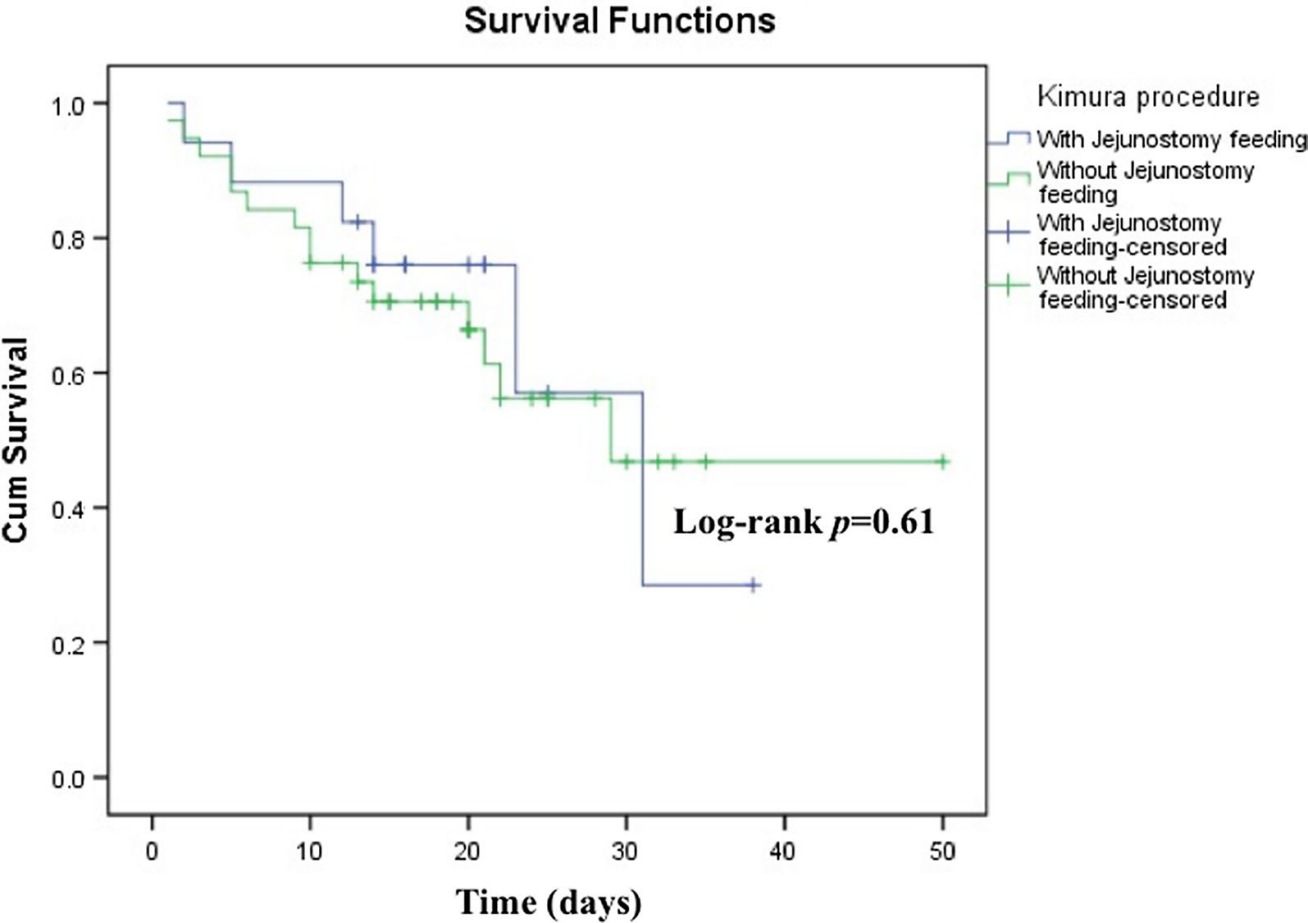
Kaplan–Meier analysis of CDO neonates’ survival after Kimura procedure at Dr. Sardjito Hospital, Indonesia. The patients’ survival analysis after surgery was not significantly different between the JFT and non-JFT groups (*p* = 0.61)

However, the length of stay following surgery was not significantly different between groups (16 [IQR, 14–22] vs. 20 [IQR, 17–28] days; *p* = 0.22) (Table 3). Moreover, overall survival of patients with CDO was 61.5%, while the survival rates of the JFT and non-JFT groups were 66.7% and 59.5%, respectively (*p* = 0.63; Table 3). The median ages at death for the JFT and non-JFT groups were 14 (12–23) and 10 (5–17) days, respectively (*p* = 0.204; Table 3). In addition, log-rank test also indicated that overall patient survival did not significantly differ between the JFT and non-JFT patients (*p* = 0.61) (Fig. 1).

### Outcomes of neonates with CDO after Kimura procedure according to patients’ sex

Next, we performed subgroup analysis of outcomes of neonates with CDO after Kimura procedure according to patients’ sex. None of the outcomes were significantly different statistically between the JFT and non-JFT groups either among male or female neonates (Table 4).

**Table 4:** Outcomes of neonates with CDO after Kimura procedure according to patients’ sex

Outcomes	Male			Female		
	JFT (n = 13)	Non-JFT (n-14)	*p* value	JFT (n = 2)	Non-JFT (n = 23)	*p* value
	Median (IQR)	Median (IQR)		Median (IQR)	Median (IQR)	
Initial enteral feeding (days)	1 (1–4)	4.5 (0.8–6)	0.23	2.5 (2.25–2.75)	5 (4–6)	0.08
Full oral feeding (days)	12 (0–15)	0 (0–9)	0.17	14 (13–15)	15 (10.5–20.5)	0.69
Duration of nasogastric output (days)	5 (4–6)	5.5 (3.25–12)	0.70	9 (9–9)	5 (4–7.5)	0.09
Length of stay (days)	16 (14–21)	13.5 (9.25–20.75)	0.41	27 (21.5–32.5)	20 (14.5–25)	0.48
Duration of parenteral nutrition (days)	13 (10–19)	11.5 (9.25–20.25)	0.77	12.5 (11.25–13.75)	17 (11.5–20)	0.39
Weight on discharge (gram)	2560 (1900–2790)	2416 (2224–2711)	0.79	2765 (2662.5–2867.5)	2250 (2015–2795)	0.23

*CDO* congenital duodenal obstruction, *JFT* jejunostomy feeding tube, *IQR* interquartile range, *N/A* not applicable

## Discussion

We are able to show the beneficial effect of JFT in neonates with CDO after the Kimura procedure regarding time to full oral feeding and duration of parenteral nutrition. Our findings were similar with a previous study [9], although they used transanastomosis nasojejunal tube (TAT) not JFT. Moreover, our study revealed that the JFT group achieved earlier enteral feeding than non-JFT feeding. It has been shown that early enteral feeding is very important for patients with CDO after surgery for wound healing and general well-being [12, 13]. Furthermore, enteral feeding also has improved the bowel peristalsis after anastomosis and reduced the complications due to parenteral nutrition [9]. Other beneficial effects of enteral feeding compared with parenteral nutrition are maintaining intestinal motility and integrity, shortening functional ileus after surgery, avoiding bacterial translocation and intestinal mucosal atrophy [9]. In addition, because of the many advantages in bowel function, enteral feeding may reduce the length of stay following surgery [9]. Interestingly, one previous study showed that patients who underwent only the Kimura procedure achieved a full oral feeding in significantly less time compared with those with Kimura procedure accompanying with JFT [11].

Several methods accompanying Kimura procedure to achieve an early enteral feeding have been reported, such as transanastomosis nasojejunal tube (TAT), post-anastomosis JFT, or gastrostomy tube [10–16]. They all have some advantages and disadvantages [10–16]. For uses of jejunal feeding tubes, these are usually divided into two methods: through a TAT [10, 13] or a post-anastomosis per-cutaneous transperitoneal JFT [11]. To the best of our knowledge, all previous studies [10, 12–16], except one [11], applied the TAT and not JFT for their CDO infants during Kimura surgery. Our study is the most current report of JFT use for the CDO infants during the Kimura procedure after the first study of its application more than 20 years ago [11]. Moreover, we compared the effectiveness of JFT vs. non-JFT for CDO infants, while one previous study [11] analyzed the efficacy of Kimura with JFT vs. duodenoduodenostomy (side to side) with TAT vs. Kimura only.

Our study showed that the length of stay and overall survival were not affected by the use of JFT during Kimura surgery. These findings were similar with a meta-analysis study by Wang et al. [14]. They also revealed that compared to TAT, JFT has lower risk for tube dislodgment, but higher risk for tube leakage [14]. In addition, length of stay and time to achieve an enteral feeding were shorter in TAT than JFT [14]. However, there are several differences between our study and Wang et al. [14]: 1) their inclusion criteria were patients ≥ 18 years of age (vs. neonates); 2) they involved patients with esophagectomy, gastrectomy, and pancreaticoduodenectomy (vs. Kimura procedure); and 3) they compared between TAT and JFT (vs. with and without JFT). Therefore, it is important to compare the outcomes of neonates with CDO following Kimura surgery accompanying with TAT vs. JFT.

Moreover, the median age at death of our patients was < 30 days, while the mortality rate in our study was 38.5% (Table 3). It might be due to the fact that most of our patients were referred late from other hospitals, and were already in severe sepsis. The mortality rate of infants with CDO varies among studies with approximately 6–58% [17–19]. Most mortality is related to sepsis as in our study [19]. Interestingly, the survival rate of infants with CDO has improved over recent decades, particularly in western countries which is associated with advances in prenatal diagnosis [19].

In our study, the JFT placement during the time of Kimura procedure was chosen according to our pediatric surgeons’ preference. These facts may affect the skewed sex distribution between groups, which was significant. Therefore, it is necessary to perform a study in the future with randomized treatments that the infants receive to clarify and confirm our findings. In addition, the insertion of a JFT might represent an additional risk for the patients, such as tube leakage [14], which might have affected the course for these patients. Our study did not encounter any complications of the JFT method in our patients with CDO.

Most of our CDO infants (64.7%) showed a polyhydramnios or double bubble during an antenatal sonography. This finding was higher than a previous report (23.3%) [16], but similar to the result (59%) in a study by Bethell et al. [3]. However, only 32.7% of our patients had complete data on antenatal sonography.

Our study revealed that most neonates with CDO were identified with Down syndrome (40.4%) and CHD (26.9%). Previous study described similar findings concerning associated anomalies in neonates with CDO [2–5]. Chen et al. [17] described the incidence of CHD in neonates with CDO which is also similar to our findings (31.72%), however, the Down syndrome was found in only 3.1% of patients. It should be noted that the differences in the distribution of congenital associated anomalies between groups in our study was statistically insignificant (Table 2). This finding might be due to the small numbers in our sample size and all associated disorders were lumped together. Although not statistically significant, these differences (Down syndrome: ~ 50% vs. ~ 30% and CHD: ~ 30% vs. ~ 20% for non-JVT vs. JFT group, respectively) should be considered during the interpretation of our findings. Moreover, it should be noted that infants without congenital anomaly received a JFT procedure twice as much as infants with congenital anomaly (9/23 vs. 6/29) (Table 2), implying the reduced loading of comorbidities in the JFT group. These facts might be related to the better outcome in the JFT group. Other facts should be considered during the interpretation of our results, including: 1) more clinical signs in the non-JFT group (*i.e.* epigastric distention; *p* = 0.024) (Table 2); and 2) a higher age at Kimura procedure in the non-JFT group, although not statistically significant (*p* = 0.103; Table 1). These findings might be associated with the worse outcome in the non-JFT group.

For the CDO etiology, annular pancreas was the most common cause found (63.4%) during the surgery in our patients. Jiang et al. [9] revealed that 66% of patients with CDO were caused by annular pancreas, while Bairdain et al. [12] reported 63% of patients with CDO had a duodenal atresia. Chen et al. [17] reported that most common CDO etiology is malrotation (53.7%) followed by annular pancreas (21.6%) and duodenal web (15%).

Notably, our findings should be interpreted very cautiously because of the small sample size from a single institution and the heterogeneity of the preferred surgical methods based on the discretion of the surgeon. In addition, there was a sex imbalance between the two groups (*i.e.* only two female neonates in the JFT group) which might be considered as an important confounding variable and affect our findings, becoming another weakness of our study.

## Conclusions

Jejunostomy feeding tube shows a beneficial effect on the time to full oral feeding, duration of parenteral nutrition and early enteral feeding in neonates with congenital duodenal obstruction after Kimura procedure.

## Data Availability

All data generated or analyzed during this study are included in the submission. The raw data are available from the corresponding author on reasonable request.

## Abbreviations

CDO: Congenital duodenal obstruction
IQR: Interquartile range
JFT: Jejunostomy feeding tube
TAT: Transanastomosis nasojejunal tube

## References

[1] Applebaum H, Sydorak R, Coran AG (2012). Duodenal atresia and stenosis-annular pancreas. Pediatric surgery.

[2] Escobar MA (2004). Duodenal atresia and stenosis: long-term follow-up over 30 years. J Ped Surg.

[3] Bethell GS, Long AM, Knight M, Hall NJ, BAPS-CASS (2019). Congenital duodenal obstruction in the UK: a population-based study. Arch Dis Child Fetal Neonatal Ed..

[4] Ein SH, Palder SB, Filler RM (2006). Babies with esophageal and duodenal atresia: a 30-year review of a multifaceted problem. J Ped Surg.

[5] Castle SL, Mathuria BJ, Torres MB (2011). Right-sided congenital diaphragmatic hernia, hepatic pulmonary fusion, duodenal atresia, and imperforate anus in an infant. J Ped Surg.

[6] Zuccarello B, Spada A, Centorrino A (2009). The modified Kimura's technique for the treatment of duodenal atresia. Int J Pediatr.

[7] Chung PH, Wong CW, Ip DK, Tam PK, Wong KK (2017). Is laparoscopic surgery better than open surgery for the repair of congenital duodenal obstruction? A review of the current evidences. J Pediatr Surg.

[8] Zingg W, Tomaske M, Martin M (2012). Risk of parenteral nutrition in neonates: an overview. Nutrients.

[9] Jiang W, Lv X, Xu X (2014). Early enteral nutrition for upper digestive tract malformation in neonates. Asia Pacific J Clin Nutr.

[10] Arnbjornsson E, Larsson M, Finkel Y, Karpe B (2002). Transanastomotic feeding tube after an operation for duodenal atresia. Eur J Ped Surg.

[11] Upadhyay V (1996). Duodenal atresia: a comparison of three modes of treatment. Eur J Ped Surg.

[12] Bairdain S (2014). A modern cohort of duodenal obstruction patients: predictors of delayed transition to full enteral nutrition. J Nutr Metab.

[13] Yhoshu E, Mahajan JK (2017). Use of simultaneous nasogastric and nasojejunal tubes for proximal intestinal atresias: a preliminary report. J Neonat Surg.

[14] Wang L, Tian Z, Liu Y (2017). Nasoenteric tube versus jejunostomy for enteral nutrition feeding following major upper gastrointestinal operations: a meta-analysis. Asia Pac J Clin Nutr.

[15] Hall NJ, Drewett M, Wheeler RA (2017). Trans-anastomotic tubes reduce the need for central venous access and parenteral nutrition in infants with congenital duodenal obstruction. Pediatr Surg Int.

[16] Harwood R, Horwood F, Tafilaj V (2019). Transanastomotic tubes reduce the cost of nutritional support in neonates with congenital duodenal obstruction. Pediatr Surg Int.

[17] Chen QJ (2014). Congenital duodenal obstruction in neonates: a decade's experience from one center. World J Pediatr.

[18] Zamir N, Akhtar J (2013). Neonatal duodenal obstruction: clinical presentation and outcome. J Surg Pakistan (Int).

[19] Rattan KN, Singh J, Dalal P (2016). Neonatal duodenal obstruction: a 15-year experience. J Neonatal Surg.

